# Unraveling Cervical Pregnancy: A Comprehensive Case Study and Future Directions

**DOI:** 10.7759/cureus.100010

**Published:** 2025-12-24

**Authors:** Juncheng Zhang, Lixian Wu, Mengmeng Yu

**Affiliations:** 1 Gynecology Department, Gansu Maternal and Child Health Care Hospital (Gansu Provincial Central Hospital), Lanzhou, CHN

**Keywords:** cervical pregnancy, ectopic pregnancy, hysteroscopy, laparoscopic exploration, temporary ligation of the uterine artery

## Abstract

Cervical pregnancy represents an uncommon form of ectopic pregnancy, characterized by its distinctive implantation site, which poses significant challenges for clinical diagnosis and management. This article examines a case study of cervical pregnancy to investigate its epidemiological characteristics, pathogenesis, clinical manifestations, diagnostic methodologies, therapeutic strategies, prevailing controversies, and prospective advancements. The objective is to furnish a clinical reference framework to inform research progress and enhance the clinical management of cervical pregnancy.

## Introduction

Cervical pregnancy is a rare form of ectopic pregnancy characterized by the implantation and development of a fertilized egg within the endocervical canal, below the histological internal os. It occurs in approximately 1 in 18,000 pregnancies, comprising fewer than 1% of all ectopic pregnancies. Despite its rarity, cervical pregnancy poses a significant risk of severe hemorrhage due to its distinctive pathophysiological mechanism, which involves embryo implantation in the cervical canal, an area with a rich blood supply and primarily fibrous connective tissue, containing only about 15% muscle content [1]. Traditional treatment approaches have resulted in a hysterectomy rate as high as 40% [2]. Recent increases in intrauterine procedures have led to a rise in the incidence of cervical pregnancy, making it a critical concern for obstetricians and gynecologists [2]. Consequently, understanding the pathogenesis and developing novel treatment strategies for cervical pregnancy have become pressing issues. This article aims to further investigate the pathogenesis and emerging treatment concepts for cervical pregnancy through the analysis of a specific case.

## Case presentation

**Figure 1:**
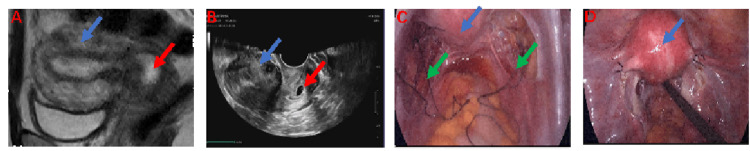
Medical imaging and surgical photographs illustrating the implications of cervical pregnancy. (A) Cervical pregnancy visualized through pelvic MRI. (B) Cervical pregnancy observed via vaginal ultrasound. (C) A laparoscopic view of temporary bilateral uterine artery ligation. (D) Postoperative images following bilateral tubal sterilization. In these images, the blue arrow indicates the uterus, the red arrow denotes the cervical pregnancy, and the green arrow marks the site of uterine artery ligation.

**Figure 2:**
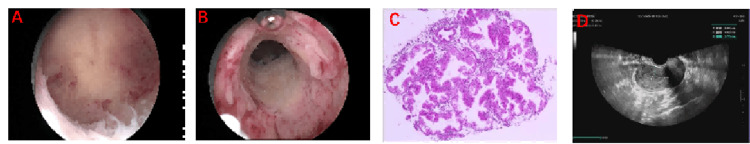
(A) Visualization of the uterine cavity via hysteroscopy following curettage. (B) Examination of the cervical canal via hysteroscopy post-uterine curettage. (C) Histological image of cervical pregnancy tissue at 20× magnification. (D) Ultrasound assessment of the uterus and cervix conducted one month post-surgery.

**Figure 3:**
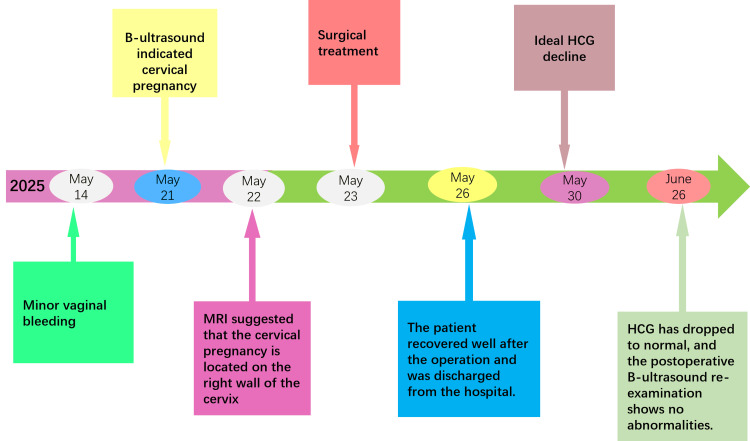
Flowchart illustrating patient diagnosis and treatment. HCG = human chorionic gonadotropin

A 38-year-old female patient, gravida 2 para 1, with a history of a cesarean section performed five years prior and regular menstrual cycles, presented with 38 days of amenorrhea accompanied by eight days of light brown vaginal bleeding. Her last menstrual period commenced on April 14, 2025. A self-administered urine pregnancy test conducted on May 14, 2025, yielded a positive result. The patient reported no abdominal pain or systemic symptoms. On May 21, 2025, a transvaginal color Doppler ultrasound identified a gestational sac located entirely within the cervical canal, lacking surrounding myometrium, which strongly suggested a diagnosis of cervical pregnancy. Subsequent pelvic MRI corroborated the presence of a 1.9 × 1.2 × 1.8 cm cystic lesion within the endocervical canal, exhibiting mixed signal intensity consistent with cervical pregnancy, and minimal pelvic effusion was also noted (Figures 1A, 1B). Upon physical examination at admission, the external genitalia appeared normal, the vagina was unobstructed, and the cervix was smooth and hypertrophied. The uterus was anteverted, of normal size and contour, with good mobility and no nodules, and the adnexa were non-tender and non-thickened bilaterally. The preoperative serum human chorionic gonadotropin (hCG) level was measured at 9,845 mIU/mL. On May 23, 2025, the patient was subjected to a laparoscopic temporary occlusion of the bilateral uterine arteries, followed by a hysteroscopic resection of the gestational tissue and concurrent bilateral tubal sterilization (Figures 1C, 1D; Figures 2A-2C). Postoperatively, the patient’s HCG levels exhibited a steady decline, normalizing within one month (Figure 3). Subsequent follow-up transvaginal ultrasound revealed no residual abnormalities in the uterus or bilateral adnexa (Figure 2D), thereby confirming the complete resolution of the ectopic pregnancy, as detailed in the diagnostic and therapeutic timeline (Figure 3).

## Discussion

Epidemiological characteristics and risk factors of cervical pregnancy

Cervical pregnancy is an uncommon form of ectopic pregnancy characterized by the implantation of a fertilized egg within the cervical canal, accounting for approximately 0.15% to 1% of all pregnancies, with an incidence rate of about 1 in 9,000 to 18,000 pregnancies [1]. The primary risk factors associated with cervical pregnancy include a history of multiple pregnancies, miscarriages, and uterine curettage [2]. High-risk groups for cervical pregnancy comprise individuals with a history of cervical surgery, such as conization or loop electrosurgical excision procedure, a history of multiple abortions (with a risk increase of 30% for three or more abortions), and intrauterine adhesions [2]. A study involving 91,067 ongoing pregnancies across 25 private assisted reproductive clinics in Spain reported an incidence of cervical pregnancy at 3.5 per 10,000 pregnancies, representing 2.02% (32 out of 1,582) of all ectopic pregnancies [2]. The study identified significant risk factors for cervical pregnancy, including having two or more prior pregnancies (odds ratio (OR) = 2.68; confidence interval (CI) = 1.18-6.07), two or more previous miscarriages (OR = 4.21; CI = 1.7-10.43), at least one previous uterine curettage (OR = 3.99; CI = 1.67-9.56), two or more previous uterine curettages (OR = 4.71; CI = 1.19-18.66), and smoking (OR = 2.82; CI = 1.14-6.94). A history of cesarean section and tubal pregnancy was not found to be associated with an increased risk of cervical pregnancy. A separate retrospective analysis of cervical ectopic pregnancy (CEP) cases revealed that, in comparison to patients with intrauterine pregnancies (IUPs), those with CEP had a significantly higher incidence of elective abortion, curettage, and cervical resection, with a substantial effect size (>0.7). When compared to patients with ectopic tubal pregnancies (TEP), CEP patients similarly exhibited a higher history of curettage and cervical resection, with a notable effect size (>0.7). The risk associated with a history of curettage was significantly greater for CEP compared to IUP (adjusted OR = 1.4; 95% CI = 1.1-9.1; p = 0.04) and TEP (adjusted OR = 6.1; 95% CI = 1.8-21.2; p = 0.04) [3]. There are regional disparities in the incidence and management of cervical pregnancy, particularly in developing countries, where the diagnosis and management of cervical pregnancy present numerous challenges. The absence of advanced diagnostic tools and techniques often leads to delayed identification of cervical pregnancies, resulting in severe maternal complications such as massive hemorrhage [4].

Pathogenesis and pathophysiological definition of cervical pregnancy

The Molecular Basis of Abnormal Embryonic Implantation

Chronic cervicitis and multiple uterine procedures, such as induced abortion and curettage, result in injury to the cervical mucosa. This injury leads to the upregulation of local inflammatory factors, including interleukin-6 and tumor necrosis factor-alpha, which compromise the defensive function of the cervical mucus plug, thereby facilitating the retention of the fertilized egg within the cervical canal [5]. Additionally, pathogen infections, such as those caused by *Chlamydia trachomatis* and *Neisseria gonorrhoeae*, activate inflammation through the TLR4/NF-kappa B signaling pathway, promoting the expression of adhesion molecules like intercellular adhesion molecule 1, which enhances embryo adhesion [6].

Dysregulation of Key Signaling Pathways

The aberrant activation of the PI3K/AKT/mTOR signaling pathway is posited to play a critical role in the sustained survival of embryos in cases of cervical pregnancy. Initially, this abnormal activation results in increased AKT phosphorylation within cervical cells, thereby promoting cellular proliferation and inhibiting apoptosis. This process contributes to the formation of a decidual-like microenvironment that is favorable for embryo survival [7]. Such dysregulation not only impacts the fundamental biological functions of cells but may also enhance the invasive capacity and metabolic activity of cells, further supporting the persistence of embryos in the cervical region [8]. Moreover, the aberrant activation of the PI3K/AKT/mTOR pathway may interact with other signaling pathways, exacerbating the pathological progression of cervical pregnancy. For instance, the interaction between the Wnt/β-catenin pathway and the PI3K/AKT/mTOR pathway is believed to be pivotal in regulating endometrial receptivity and embryo implantation. Dysregulation in any of these pathways may result in implantation failure or adverse pregnancy outcomes [9].

Uterine Structural Abnormalities

Uterine malformations, such as a septate uterus, can disrupt the expression of the *HOXA10* and *HOXA11* genes. These genes are crucial for the normal functioning of the endometrium, with their expression being modulated by estrogen and progesterone, peaking during the secretory phase of the menstrual cycle. *HOXA10* and *HOXA11* facilitate endometrial differentiation and embryo implantation by regulating extracellular matrix remodeling, the expression of cell adhesion molecules such as β3-integrin, and the secretion of cytokines such as leukemia inhibitory factor. Additionally, they may interfere with the normal directional migration of embryos [10].

Exploration of the gold standard for diagnosis

The diagnosis of cervical pregnancy primarily relies on clinical manifestations, ultrasound examination, and laboratory tests. Early and accurate identification of cervical pregnancy is crucial for developing appropriate treatment strategies and enhancing patient outcomes. Currently, ultrasound serves as the principal modality for the early diagnosis of cervical pregnancy. Transvaginal ultrasound effectively delineates the location, size, shape, and spatial relationship of the cervical pregnancy with adjacent tissues, thereby providing a critical foundation for diagnosis. With technological advancements, three-dimensional ultrasound offers more comprehensive imaging data, facilitating a more precise assessment of the pregnancy. For instance, in diagnosing cervical pregnancy, three-dimensional ultrasound can more effectively illustrate the relationship between the gestational sac and the cervical canal, thereby improving diagnostic accuracy [11]. MRI holds significant value in diagnosing cervical pregnancy, particularly in complex cases or those challenging to diagnose via ultrasound. MRI offers detailed soft tissue information that aids clinicians in determining the precise location of the pregnancy and placental implantation. Research has documented MRI findings of CEP during the second trimester, including the presence of the fetus within the cervix, endometrial hyperplasia with an empty uterine cavity, and irregular placental implantation in the cervix. Accurate identification of these features is crucial to prevent severe hemorrhage [12]. Furthermore, elastography can evaluate the elasticity of cervical tissue, providing insights into cervical maturity and firmness, which are associated with pregnancy outcomes. This technique holds potential significance in predicting preterm birth or successful labor induction; however, there remains no consensus on the optimal assessment method [13]. Previous research has utilized loop-mediated isothermal amplification in conjunction with machine learning techniques on bovine vaginal mucosa to facilitate early pregnancy detection. This approach offers a novel methodology for early pregnancy diagnosis by targeting the expression of specific genes. Nonetheless, further investigation is required to explore the applicability of this technology in diagnosing cervical pregnancy [14].

Cervical pregnancy treatment

Various treatment modalities are available for cervical pregnancy, including pharmacological interventions, surgical procedures, and combination therapies, each with distinct advantages and limitations. Methotrexate (MTX) is frequently employed in pharmacological treatment due to its ability to inhibit trophoblastic cell proliferation, disrupt villous structures, and induce necrosis, exfoliation, and resorption of embryonic tissue. Among surgical options, uterine artery embolization (UAE) followed by immediate curettage has been documented. A study involving 16 patients with cervical pregnancy reported successful outcomes in 15 patients using this approach, which effectively facilitated the regression of serum hCG levels and cervical mass, preserved fertility, and reduced hospital stay duration [15]. Comparative studies have evaluated various treatment strategies, such as systemic MTX infusion, UAE, and their combination, for managing CEP. Findings indicate that the combined treatment group experienced no severe complications, exhibited a rapid decline in serum β-hCG levels, and demonstrated shortened menstrual recovery time. Minimally invasive surgical techniques offer enhanced treatment options for cervical pregnancy, characterized by reduced trauma and expedited recovery. Hysteroscopy is a frequently employed minimally invasive procedure that allows for direct visualization and resection or ablation of the gestational tissue within the cervix. For instance, a study demonstrated the efficacy of a two-step hysteroscopic approach in managing cervical pregnancy. Initially, a 5-mm hysteroscope was utilized to confirm the diagnosis, terminate the pregnancy, and achieve partial hemostasis through vessel coagulation. Subsequently, a 10-mm hysteroscope facilitated the removal of the gestational sac and villous tissue following cervical dilation. The patient exhibited a favorable postoperative recovery, with serum β-hCG levels returning to negative within 20 days, and a subsequent normal pregnancy occurring after five months. This method has been deemed safe and effective [16]. Additionally, UAE represents a significant minimally invasive treatment modality aimed at reducing blood flow to the cervical pregnancy site and mitigating the risk of surgical hemorrhage through UAE. UAE is frequently employed in conjunction with curettage. A study involving 16 patients with cervical pregnancy demonstrated that an initial UAE followed by immediate curettage resulted in successful treatment for 15 patients, highlighting the benefits of this combined approach in managing hemorrhage and preserving fertility [17]. Furthermore, minimally invasive techniques, such as ultrasound-guided laser ablation and the use of a cervical mature double balloon catheter, have been shown to effectively terminate pregnancy while minimizing uterine damage and enhancing the likelihood of fertility preservation.

Pharmacological interventions are pivotal in managing cervical pregnancy, with recent advancements enhancing treatment efficacy. MTX, a conventional therapeutic agent, continues to be extensively utilized; however, its administration methods and dosage regimens are subject to ongoing optimization. Notably, the triple therapy approach, involving intra-arterial MTX infusion in conjunction with UAE followed by curettage, has demonstrated feasibility and significant benefits in treating CEP [18]. This regimen has been associated with a rapid decline in serum β-hCG levels, expedited resumption of menstruation, swift resolution of cervical mass, and reduced duration of hospitalization [19]. Some studies have tried new drug combinations, such as cervical ripening balloon combined with MTX and potassium chloride (KCl) for the treatment of CEP at 13 weeks. KCl and MTX were injected into the gestational sac first, followed by cervical ripening and double balloon placement. The balloon was removed 72 hours later, and the pregnancy was resolved after 12 weeks. It provides a new idea for the treatment of late CEP [18]. In addition, some studies have explored transvaginal injection of anhydrous ethanol (AE) for the treatment of cervical pregnancy and cesarean scar pregnancy. AE is injected into the hyperechoic ring (lacunar space) around the gestational sac under ultrasound guidance, and the serum β-hCG level is effectively reduced in most patients; some patients subsequently experienced successful pregnancy and delivery. This suggests that this method may be an effective alternative treatment [19].

Future advancements in the treatment of cervical pregnancy may include innovative approaches. One potential development is the creation of more targeted pharmacological interventions, which could arise from a deeper understanding of the pathological mechanisms underlying cervical pregnancy. For instance, drugs designed to specifically target cervical gestational trophoblasts could enhance treatment efficacy while minimizing harm to healthy tissues. Additionally, advancements in surgical techniques may also emerge, further improving treatment outcomes. Furthermore, advancements in biomaterials and tissue engineering technology hold the potential to introduce innovative approaches for the management of cervical pregnancy. These advancements may include the repair of damaged cervical tissue using biomaterials, thereby facilitating the restoration of normal physiological function. Additionally, novel integrative treatment models are likely to emerge, which combine pharmacological interventions and surgical procedures with cutting-edge technologies. Such models aim to offer patients more personalized and effective treatment plans, enhance treatment success rates, and minimize the risk of complications, all the while preserving fertility.

## Conclusions

The management of cervical pregnancy may involve pharmacological and surgical interventions. MTX is frequently utilized in medical treatment, particularly for early-stage cases characterized by asymptomatic or mild symptoms, a small gestational sac, and the absence of fetal cardiac activity. Treatment may require single or multiple doses, with careful monitoring of changes in hCG levels. However, medical management can be protracted and carries the risk of failure, potentially necessitating surgical intervention. In surgical management, uterine curettage may be indicated if the patient experiences excessive bleeding or if pharmacological treatment proves ineffective. Nevertheless, uterine curettage in cervical pregnancy poses a significant risk of hemorrhage, necessitating pretreatment strategies such as UAE to mitigate intraoperative bleeding. Additionally, hysteroscopic surgery offers a more precise approach, enabling direct visualization and removal of gestational tissue, thereby minimizing trauma. In cases where hemorrhage cannot be adequately controlled, more invasive surgical interventions, such as cervical cerclage or hysterectomy, may become necessary. However, hysterectomy is typically considered a last resort, particularly for patients who do not have future fertility considerations. An alternative approach is UAE, which can be utilized preoperatively to occlude blood flow and mitigate bleeding risks, or as an emergency intervention in instances of acute hemorrhage. Postoperative monitoring of hCG levels is essential to confirm the absence of residual trophoblastic tissue and prevent persistent ectopic pregnancy. In this case, the patient presented with 38 days of amenorrhea and eight days of minor vaginal bleeding, with an hCG level of 9,845 mIU/mL. Given the continued minor vaginal bleeding and the presence of surgical indications, a procedure involving laparoscopic temporary occlusion of the uterine artery, uterine curettage, and hysteroscopy was recommended. As the patient did not have plans for future fertility, sterilization was also advised. The patient subsequently underwent laparoscopic temporary occlusion of the uterine artery, bilateral tubal sterilization, uterine curettage, and hysteroscopy, and demonstrated a favorable recovery.
